# Effect of the COVID-19 pandemic and lockdown on cancer stage distribution and time to treatment initiation using cancer registry data of the Swiss cantons of Zurich and Zug from 2018 to 2021

**DOI:** 10.1007/s00432-025-06140-x

**Published:** 2025-02-21

**Authors:** Flurina Suter, Miriam Wanner, Andreas Wicki, Dimitri Korol, Sabine Rohrmann

**Affiliations:** 1 Division of Chronic Disease Epidemiology, Epidemiology, Biostatistics and Prevention Institute (EBPI), University of Zurich, Zurich, Switzerland; 2 Cancer Registry Zurich, Zug, Schaffhausen and Schwyz, Institute of Pathology and Molecular Pathology, University Hospital Zurich, Zurich, Switzerland; 3 Department of Medical Oncology and Hematology, Faculty of Medicine, University and University Hospital Zurich, Zurich, Switzerland

**Keywords:** Coronavirus disease 2019, Cancer stage distribution, Time to treatment initiation, Switzerland, Cancer registry

## Abstract

**Purpose:**

Swiss healthcare institutions conducted only urgent procedures during the COVID-19 lockdown, potentially leading to a lack of care for other severe diseases, such as cancer. We examined the effects of the pandemic on cancer stage distribution and time between cancer diagnosis and treatment initiation using population-based cancer registry data.

**Methods:**

The study was based on data of the cancer registry of the cantons of Zurich and Zug from 2018 to 2021. Cancer stage distribution was analysed descriptively and with a Pearson’s Chi-squared test. Time between cancer diagnosis and treatment initiation was determined in days and analysed descriptively and by fitting Quasipoisson regression models.

**Results:**

For all-cancer and colorectal, lung, and prostate cancer statistically significant evidence for a difference in cancer stages distribution among the incidence years was observed. Based on the all-cancer regression models, longer time to treatment initiation (TTI) was observed for patients diagnosed in 2021 and receiving surgery (Rate Ratio = 1.08 [95% confidence interval 1.03, 1.14]) or hormone therapy (1.20 [1.03, 1.40]) compared to those diagnosed in 2018/19 receiving those therapies. We observed no difference in TTI between cancer patients diagnosed in 2020 compared to 2018/19 for any of the therapies investigated, except for chemotherapy with shorter TTI (0.92 [0.86, 0.98]).

**Conclusion:**

The observed effects on cancer outcomes in 2020 and 2021 compared to 2018/19 coincided with the beginning of the COVID-19 pandemic in Switzerland in 2020 onwards. Short- and long-term effects of the pandemic on cancer outcomes and the public healthcare system were observed. However, we cannot exclude that the implementation of the new Swiss law on cancer registration in 2020 explains part of our observations.

**Supplementary Information:**

The online version contains supplementary material available at 10.1007/s00432-025-06140-x.

## Introduction

The first coronavirus disease 2019 (COVID-19) case was detected in December 2019 in Wuhan, China (Umakanthan et al. 2020; World Health Organization and Chinese Research Team 2021). The disease is caused by the severe acute respiratory syndrome coronavirus 2 (SARS-CoV-2) that is transmitted between humans mainly through aerosols and direct contact (Umakanthan et al. 2020). On January 30, 2020, the World Health Organization (WHO) defined the outbreak as a Public Health Emergency of International Concern (World Health Organization 2020). Due to the rapid spread and the disease severity, the WHO announced on March 11, 2020, the COVID-19 pandemic (World Health Organization 2020).

In Switzerland, the first confirmed COVID-19 case was reported on February 25, 2020 (Federal Office of Public Health 2020a). Shortly thereafter, Switzerland mandated a national lockdown of public life from March 17 to April 26, 2020, to decrease the number of new COVID-19 infections (Federal Office of Public Health 2020b). During the lockdown phase no public or private events were allowed, shops, restaurants, bars, and entertainment and leisure facilities had to close, people were advised to keep distance, to avoid unnecessary contacts, and, especially elderly people, to stay at home (The Federal Council 2020). Healthcare institutions were allowed to conduct only urgent procedures (The Federal Council 2020), potentially leading to a lack of care for other severe diseases, such as cancer.

Due to the lockdown, cancer screening programs in many European countries, including Switzerland, were interrupted (Swiss Cancer Screening 2021; Neamţiu et al. 2022). Effects of the pandemic and the lockdown on cancer outcomes were observed in manifold ways, such as fewer reported diagnoses (Dinmohamed et al. 2020; Kaufman et al. 2020; Peacock et al. 2021; Ribes et al. 2022; Mentrasti et al. 2022; Heer et al. 2023; Mostafavi Zadeh et al. 2023; Trojanowski et al. 2023; Angelini et al. 2023; Bennett et al. 2024), an upshifting in stage distribution (Radulovic et al. 2021; Meerwein et al. 2021; Rottoli et al. 2022; Davis et al. 2022; Kaltofen et al. 2022; Kostner et al. 2022; Mentrasti et al. 2022; Heer et al. 2023; Peacock et al. 2023, 2024; Bennett et al. 2024; Triki et al. 2024; Clements et al. 2024), and prolonged time between diagnosis and first treatment (Tasoulas et al. 2023; Triki et al. 2024). Depending on the cancer type and country, the effects on cancer outcomes ranged from unfavourable (Mostafavi Zadeh et al. 2023; Angelini et al. 2023; Triki et al. 2024; Clements et al. 2024) to no effects (Hawrot et al. 2021; Parikh et al. 2022; Keogh et al. 2022; Feron Agbo et al. 2023) or even more beneficial circumstances (Mendonça e Silva et al. 2023; Rasic et al. 2023; Filipas et al. 2024) during COVID-19 compared to pre-COVID-19.

To the best of our knowledge, the effects on cancer care delivery in Switzerland have been evaluated, to some extent, only by a few previous studies (Meerwein et al. 2021; Kostner et al. 2022; Suter et al. 2024). In Switzerland, a lack of knowledge about overall cancer stage distribution and time between cancer diagnosis and initiation of first treatment exists. Especially studies based on population-based and not clinical-based cancer data are missing in Switzerland. Therefore, the aim of the current study was to investigate the effects of the COVID-19 pandemic and lockdown on cancer stage distribution and time to first treatment initiation (TTI) by using population-based cantonal cancer registry data of the cantons of Zurich (ZH) and Zug (ZG) from 2018 to 2021, reflecting short-term but also long-term potential impacts of the pandemic. The findings of this study aim to provide insight for policy makers and healthcare institutions on healthcare delivery during a pandemic to increase preparedness for a future COVID-19 wave or pandemic.

## Methods

The structure of the REporting of studies Conducted using Observational Routinely-collected Data (RECORD) guidelines was followed (Nicholls et al. 2015).

### Cancer registry and study population

Even though the first Swiss canton started cancer registration already in 1969 (Basel Cancer Registry 2023), cancer registration was not mandatory in Switzerland until 2020, when a new national law came into force (Federal Office of Public Health 2022). The law ensures a nationwide, complete, detailed, and high-quality documentation of all cancer cases by the cantonal cancer registries (Federal Office of Public Health 2022). For the current study, data from the cantonal cancer registry of the Swiss cantons of ZH, ZG, Schaffhausen (SH), and Schwyz (SZ) (CRZZSS), to which the study investigators had full access, were used. A detailed overview of the data registration process at the CRZZSS including data collection, data linkage, and data cleaning is available online (CRZZSS 2023). In 2018, before the implementation of the new Swiss law, data quality of the CRZZSS has been investigated with respect to completeness, comparability, timeliness, and validity and was stated as good (Wanner et al. 2018).

The cantons of ZH and ZG started registration in 1980 and 2011, whereas the cantons of SZ and SH started registration in 2020. Because we used data from 2018 to 2021, only data of residents with a cancer diagnosis living in the cantons of ZH and ZG were used in the current study. Both cantons have cancer incidence rates comparable to the national rates (CRZZSS 2024; National Agency for Cancer Registration 2024). In comparison to other cantons, the cantons of ZH and ZG do not have a cantonal cancer screening program implemented which could have been interrupted during the pandemic (Swiss Cancer Screening 2021).

For each cancer case, the CRZZSS documented detailed information on the patient, such as age, sex, canton of residence, and on the cancer, such as incidence date, indicating the date of cancer diagnosis based on the definitions by the National Agency for Cancer Registration and the Childhood Cancer Registry (Staehelin et al. 2025), first treatment date, stage classification based on the international classification of diseases (10th revision) (ICD-10) (World Health Organization 2019) code, and TNM stage based on the TNM classification of malignant tumors, 8th edition, by the Union of International Cancer Control (Union of International Cancer Control 2016).

For the analysis on cancer stage distribution, only cases of a malignant, solid tumour were included (except non-melanoma skin cancer ICD-10: C44). In our study, we excluded non-melanoma skin cancer cases because these cases were registered at the CRZZSS with the implementation of the cancer registration law in 2020. Therefore, reliable and representative data on non-melanoma skin cancers were not available for the pre-pandemic period (2018/19). Cancer stage at diagnosis was defined based on the pathological TNM stage or, if the pathological TNM stage was unavailable, on the clinical TNM stage. The American Joint Committee on Cancer tumour, node, and metastasis stage classification manual was used to categorize cases as stage I, II, III, IV, or unknown (Amin et al. 2016). A cancer case in our data can be of ‘unknown’ stage if e.g., healthcare institutions did not forward the information to the cancer registry or the medical diagnostic tests to define cancer stage were not conducted due to personal reasons or comorbidities of the patient.

For the analysis on TTI all malignant tumours (except non-melanoma skin cancer ICD-10: C44) and benign brain tumours (ICD-10: D32-33, D43) were included. For the classification of cancer treatment, the Swiss Classification of Surgical Procedures codes (CHOP codes; Version 2021) (Federal Statistical Office 2023) were used, which are updated by the Federal Statistical Office on an annual base. CHOP codes were categorised into seven treatment groups: surgery, radiotherapy, chemotherapy, immunotherapy, hormone therapy, stem cell transplant, and other treatment. The latter treatment category included every other type of conducted treatment including, among other therapies, active surveillance, targeted therapies, and hyperthermia therapies. TTI was defined as the difference in days between the date of cancer diagnosis and the date of the first treatment for the respective cancer. Further therapies conducted after the first therapy were not investigated in our study due to unavailability of the corresponding data. Cancer cases were excluded if the CHOP code for cancer therapy was missing, therapy was coded as ‘unknown’ or ‘none’, therapy start date was missing, TTI was ≥ 300 days, or any additional incident cancer diagnosis following the first primary cancer within the period 2018–2021.

In the analyses on cancer stage distribution and TTI, the five most frequent cancer types in Switzerland were investigated individually: colorectal cancer (ICD-10: C18-C20), lung cancer (ICD-10: C34), skin melanoma (ICD-10: C43), female breast cancer (ICD-10: C50), and prostate cancer (ICD-10: C61).

A flowchart of the study population for each analysis with more detailed information on the exclusion criteria and the excluded patients is provided in the supplementary materials (Figure S1).

### Statistical analyses

Baseline characteristics of the study population were analyzed descriptively. The distribution of cancer stage stratified by incidence year is displayed in contingency tables in absolute numbers and percentages. The latter percentages are additionally visualized in Figures, displaying the percentage of unknown staged cancer cases separately. A Pearson’s Chi-squared test was used to test for independence of cancer stage and cancer incidence year. In addition, multivariable multinomial regression models were fitted to further investigate the association between cancer stage distribution and incidence years. The models were adjusted for incidence year (2018/19, 2020, 2021) and month (January to December) and the patient’s sex (male, female), age group (<60, 60-69, 70-79, >=80 years of age), and canton of residence (ZH, ZG).

The absolute number and percentage of conducted treatments stratified by incidence year are displayed in a contingency table. TTI stratified by incidence year and treatment group was descriptively analyzed and visualized using boxplots. To investigate the association between incidence year and TTI, a Quasipoisson regression model was fitted for each treatment group and cancer type separately and the corresponding 95% confidence intervals (95% CI) were calculated. For each cancer type only treatment groups with at least ten cases per incidence year were analyzed. The outcome was TTI counted in days. As explanatory variables, incidence year (2018/19, 2020, 2021), incidence month (January to December), cancer stage (I to IV or unknown), patients’ sex (male, female), canton of residence (ZH, ZG), and age group (< 60, 60–69, 70–79, >=80 years of age) were used.

If a cancer case at the CRZZSS had missing information on the exact day of cancer incidence or treatment start, the CRZZSS used the 15th of the corresponding month as date. Meanwhile, if information on day and month were missing, the CRZZSS used the 30th of June of the corresponding year as date. In a sensitivity analysis, Quasipoisson regression models were fitted excluding cancer cases with an imputed incidence or treatment start date. The sensitivity analysis enabled our study to check the robustness of the findings of our main analyses, since small differences in TTI when using imputed compared to the true but unknown dates, can influence the results.

For all analyses, the software R (version 4.3.1 (R Core Team 2021) and a two-sided p-value of 0.05 for statistical significance were used.

## Results

Characteristics of cancer patients included for the analysis on cancer stage distribution are shown in Table 1, whereas the characteristics of cancer patients included for the analysis on TTI are displayed in Table S1. The study population for the analysis on TTI was slightly smaller (*n* = 28,083) than the one on cancer stage distribution (*n* = 29,973), mainly due to more restrictive exclusion criteria. Otherwise, the two study populations were comparable and therefore, only the study population on cancer stage distribution is further described. The proportion of males was larger than the proportion of females for all-cancers (52.9% versus 47.1%), colorectal cancer (51.3% versus 48.7%), lung cancer (55.8% versus 44.2%), and skin melanoma (54.3% versus 45.7%). Median age at incidence was highest for colorectal cancer (71.0 years) and lung cancer (71.0), followed by prostate cancer (70.0), all-cancer (69.0), skin melanoma (66.0), and female breast cancer (63.0). The distribution of cancer cases across incidence years was similar, except for slightly more prostate cancer diagnoses in 2021 (28.6%) compared to the previous years.

**Table 1:** Baseline characteristics of the study population on cancer stage distribution for the all-cancer and the five most common cancer types using data of the Swiss cantons of Zurich and Zug between 2018/19 and 2021

Variables	All-cancer^a^	Colorectal Cancer^a^	Lung Cancer^a^	Skin Melanoma^a^	Female Breast Cancer^a^	Prostate Cancer^a^
**n** **Sex**, n (%) Males Females	29,973 15,854 (52.9%) 14,119 (47.1%)	3291 1688 (51.3%) 1603 (48.7%)	3356 1872 (55.8%) 1484 (44.2%)	2984 1620 (54.3%) 1364 (45.7%)	4879 0 (0.0%) 4879 (100.0%)	5473 5473 (100.0%) 0 (0.0%)
**Age at Incidence** (median [IQR]) **Canton**, n (%) Zurich (ZH) Zug (ZG)	69.0 [58.0, 77.0] 27,687 (92.4%) 2286 (7.6%)	71.0 [60.0, 80.0] 3018 (91.7%) 273 (8.3%)	71.0 [63.0, 77.0] 3120 (93.0%) 236 (7.0%)	66.0 [52.0, 77.0] 2705 (90.7%) 279 (9.3%)	63.0 [51.0, 74.0] 4477 (91.8%) 402 (8.2%)	70.0 [64.0, 76.0] 5073 (92.7%) 400 (7.3%)
**Incidence Year**, n (%) 2018–2019 2020 2021	14,810 (49.4%) 7301 (24.4%) 7862 (26.2%)	1657 (50.3%) 786 (23.9%) 848 (25.8%)	1686 (50.2%) 845 (25.2%) 825 (24.6%)	1478 (49.5%) 719 (24.1%) 787 (26.4%)	2460 (50.4%) 1157 (23.7%) 1262 (25.9%)	2591 (47.3%) 1318 (24.1%) 1564 (28.6%)

IQR = Interquartile range;

^a^ Cancer cases were defined using the 10th revision of the international classification of diseases (ICD-10): All-cancer: all malignant cancers (except C44) and benign brain cancer (ICD-10: D32-33, D43); Colorectal cancer: ICD-10 C18-C20; Lung cancer: ICD-10 C34; Skin melanoma: ICD-10 C43; Female breast cancer: ICD-10 C50; Prostate cancer: ICD-10 C61 (World Health Organization 2019)

**Fig. 1:**
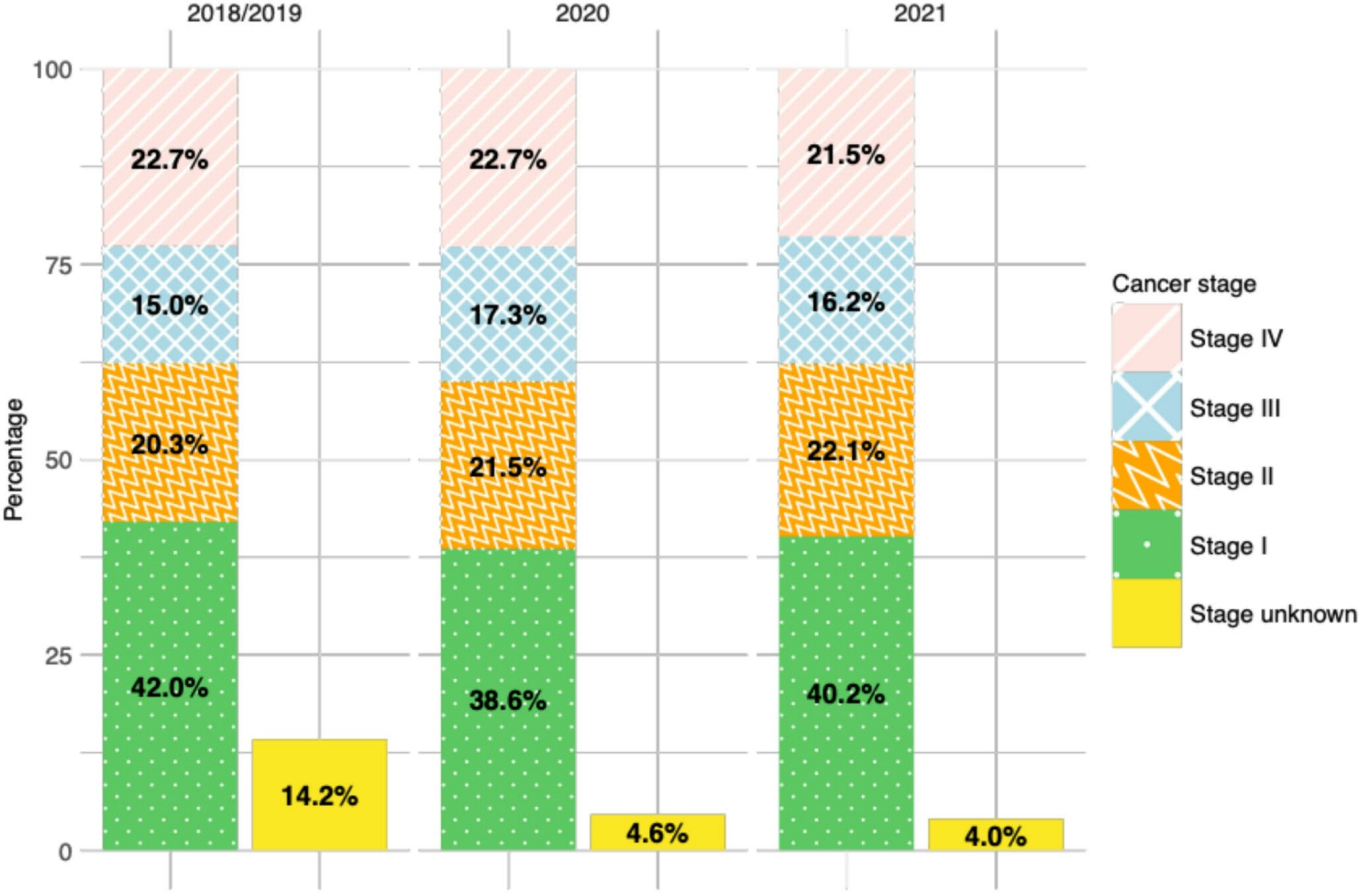
Cancer stage distribution of all solid cancer cases in the Swiss cantons of Zurich and Zug from 2018 to 2021 (*n* = 29,973)

In Fig. 1 and S2-6 the percentages are visualized in bar plots, displaying the percentage of cancer cases with unknown stage as a separate bar. For all-cancer a slight increase in stage II and III cancer cases was seen during COVID-19 compared to pre-COVID-19 (2020: +1.2 and + 2.3% points; 2021: +1.8 and + 1.2% points, respectively). Among the five most common cancer types, except skin melanoma, shifts in the distribution of stage I to IV cancer cases were observed, even though no overall trend could be determined. For cases with unknown stage, a decline in 2020 and 2021 compared to 2018/19 was seen for all-cancer (2020: -9.6% points; 2021: -10.2% points) and each of the five most common cancer types, even though much less for skin melanoma and female breast cancer. The Chi-squared test showed evidence for dependence between cancer stage and incidence year for all-cancer (χ^2^ = 732; df = 8; p-value = < 0.001), colorectal (χ^2^ = 81; df = 8; p-value = < 0.001), lung (χ^2^ = 102; df = 8; p-value = < 0.001), and prostate cancer (χ^2^ = 100; df = 8; p-value = < 0.001) but not for skin melanoma (χ^2^ = 11; df = 8; p-value = 0.23) and female breast cancer (χ^2^ = 10; df = 8; p-value = 0.24). The results of the multivariable multinomial regression models supported the descriptive findings and results of the Chi-squared tests (Table S2).

**Fig. 2:**
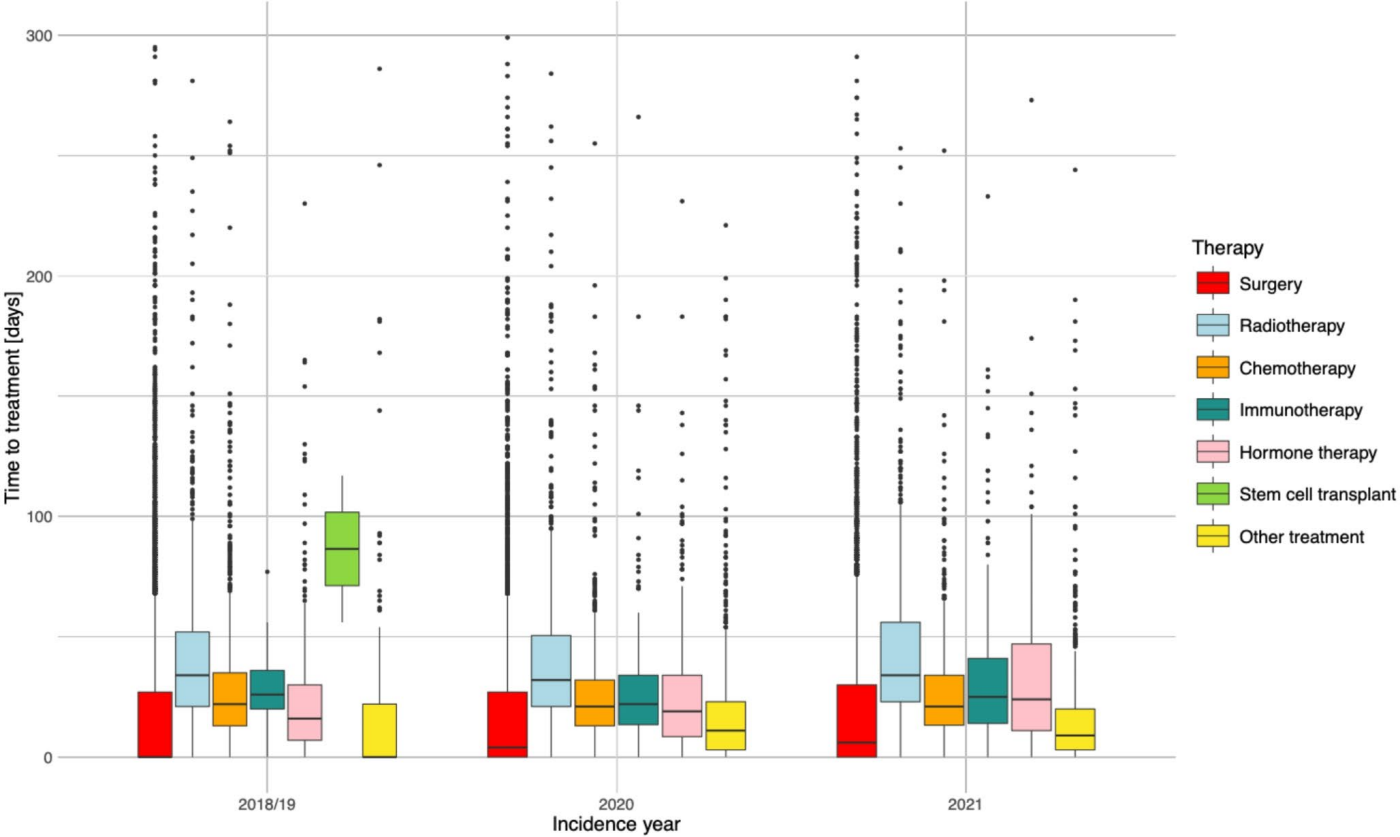
Distribution of the time to treatment initiation stratified by incidence year and treatment group for all-cancer cases from 2018/19 to 2021 in the cantons of Zurich and Zug combined (*n* = 28,083)

**Fig. 3:**
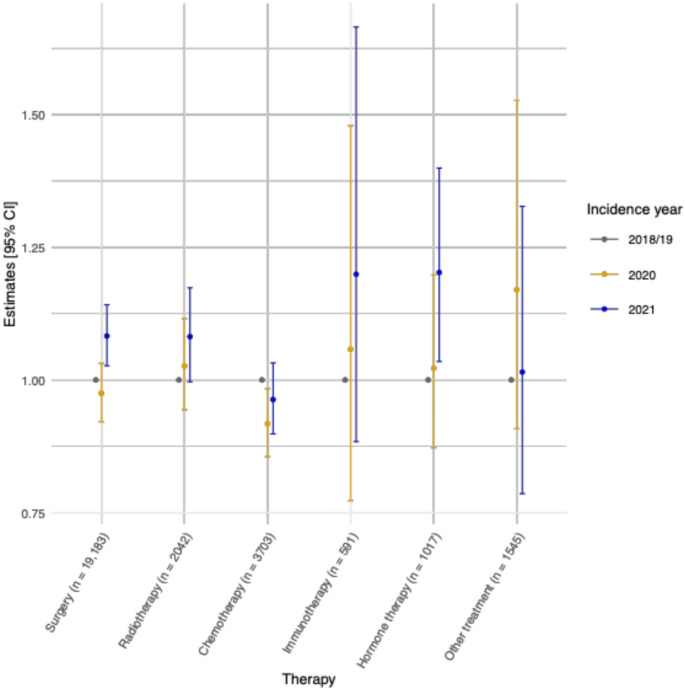
Quasipoisson regression models estimates and 95% confidence intervals of the time to treatment initiation stratified by incidence year and treatment group for all-cancer cases from 2018/19 to 2021 in the cantons of Zurich and Zug combined (*n* = 28,081)

Patients diagnosed in 2018/19 received more often surgery (75.6%), but less frequently immunotherapy (0.4%) or other treatments (1.4%) compared to patients diagnosed in 2020 (61.7%; 3.3%; 8.9%) and 2021 (62.9%; 3.8%; 8.8%; Table 2). Tables S3-7 show the corresponding distribution of patients by incidence year and treatment group for the five cancer types. A decrease of conducted surgeries was seen among all cancer types in 2020 and 2021 compared to pre-COVID-19, ranging from 0.6 to 23.6%. Compared to 2018/19, increases in immunotherapy and other treatments were observed in 2020 (+ 5.9% points; +9.2% points) and 2021 (+ 8.4% points; +8.5% points) for lung cancer, whereas increases in hormone therapy and other treatments were observed for prostate cancer in 2020 (+ 8.8% points; +11.6% points) and 2021 (+ 7.6% points; +10.2% points). For skin melanoma, breast, and colorectal cancer the difference in the percentages of conducted treatments across incidence years were the smallest.

**Table 2:** All-cancer cases in the cantons of Zurich and Zug diagnosed between 2018/19 and 2021 stratified by incidence year and type of treatment (*n* = 28,083)

	Incidence year		
	2018/19^a^ **(** *n* ** = 6318)**	2020 **(** *n* ** = 7469)**	2021 **(** *n* ** = 7979)**
**Type of treatment**, n (%)			
Surgery Radiotherapy Chemotherapy Immunotherapy Hormone therapy Stem cell transplant Other treatment	4778 (75.6%) 436 (6.9%) 825 (13.1%) 23 (0.4%) 170 (2.7%) 1 (0.0%) 87 (1.4%)	4606 (61.7%) 583 (7.8%) 1043 (14.0%) 243 (3.3%) 327 (4.4%) 0 (0.0%) 667 (8.9%)	5022 (62.9%) 588 (7.4%) 1010 (12.7%) 303 (3.8%) 351 (4.4%) 0 (0.0%) 705 (8.8%)

^a^ Average absolute number and percentage of diagnoses from 2018 to 2019

The distribution of TTI by incidence year and treatment group is displayed for all-cancer in Fig. 2 (*n* = 28,083) and for the specific cancer types in Figures S7-11. Focusing on all-cancer, the overall median TTI was 13 days in 2018/19, 14 days in 2020, and 15 days in 2021. The TTI varied between the treatment types with stem cell transplant (overall only 2 cases in 2018/19) having the largest median TTI (86.5 days), followed by radiotherapy (34 days), whereas surgery revealed the smallest median TTI (2 days) when combining the data of all incidence years. For surgery, median TTI was zero days for all-cancer patients diagnosed in 2018/19, lung cancer patients diagnosed in 2018/19 and 2021, and for skin melanoma patients in all three incidence periods.

The point estimates and 95% CIs of the Quasipoisson regression models fitted by treatment and cancer type are shown in Fig. 3 and S12-16. Evidence for shorter TTI among all-cancer patients diagnosed in 2020 compared to 2018/19 was observed for those receiving chemotherapy (Rate Ratio (RR) = 0.92 [95% CI 0.86, 0.98]). Meanwhile, statistically significantly longer TTI were observed for all-cancer patients diagnosed in 2021 compared to those diagnosed in 2018/19 if they received surgery (1.08 [1.03, 1.14]) or hormone therapy (1.20 [1.03, 1.40]; Fig. 3).

Among the five most common cancer types, no overall trend was observed. Differences in TTI during COVID-19 compared to pre-COVID-19 varied by cancer type with skin melanoma not showing any statistically significant differences among the investigated incidence years (Figures S12-16).

In the sensitivity analyses, excluding patients with an imputed cancer incidence or treatment start date, 1088 all-cancer patients were excluded of which 70 were colorectal, 141 lung, 12 skin melanoma, 151 female breast, and 258 prostate cancer cases. The results of the sensitivity analyses (Figures S18-23) were in line with the findings of the main analysis, except for the results on lung cancer. In the latter sensitivity analysis, no evidence for a difference in TTI among patients diagnosed in 2020 compared to 2018/19 and receiving surgery was present anymore. Instead, evidence was found for shorter TTI among lung cancer patients receiving chemotherapy and diagnosed in 2021 compared to those diagnosed in 2018/19 (0.80 [0.67, 0.94]).

## Discussion

The current study was based on data of patients registered at the cancer registry of the cantons of ZH and ZG from 2018 to 2021. Coinciding with the spread of the COVID-19 pandemic in Switzerland but also with the implementation of the new national cancer registration law in 2020, the cancer stage distribution generally shifted towards fewer cases of unknown stage and slightly more of stage II and III. There was evidence for differences in cancer stage distribution among incidence years for all cancers except skin melanoma and female breast cancer. During COVID-19 less surgeries and more other treatments were conducted compared to pre-COVID-19 years. Shorter TTI for chemotherapy was observed for all-cancer patients diagnosed in 2020 compared to those diagnosed in 2018/19. Meanwhile, longer TTI for surgery and hormone therapy was observed for all-cancer patients diagnosed in 2021 compared to 2018/19, indicating that there was no evidence for a significant delay in all-cancer treatment during the first, but for some therapies during the second year of the COVID-19 pandemic compared to pre-COVID-19.

The most remarkable shift in cancer stage distribution was towards fewer cancer cases of unknown stage in 2020 and 2021 compared to 2018/19. This was observed among all investigated cancers to some extent, although only marginally for skin melanoma and female breast cancer. The decrease of unknown stage cancer cases from 2020 onward is likely influenced by both the pandemic and the new national law on cancer registration. The pandemic has increased the importance of detailed disease surveillance (Assefa et al. 2021; Cardwell et al. 2023). Therefore, we speculate that this may have led to an increased awareness to report cancer cases in detail. On the other hand, the new Swiss law, obligating all private and public health institutions to report all cancer cases in detail to the cantonal cancer registries, could have been the main reason for the decrease in unknown stage cancer cases from 2020 onward. A different trend on cancer cases with unknown stage was observed in previous studies. No decrease in unknown stage all-cancer cases during COVID-19 was observed in Bavaria, Germany (Voigtländer et al. 2021). Previous studies from the UK found an increase of unknown stage all-cancer cases in 2020 compared to the pre-pandemic period by 17% points in England (Ambler and Lowes 2022) and of unknown staged breast and colorectal cancer cases in Wales by 55.8% points and 803.6% points (Greene et al. 2022), respectively.

In the current study, slightly more cancer cases of stage II and III were observed during the pandemic compared to pre-pandemic. The latter increase might reflected the hesitation of people to visit public healthcare institutions due to the national pandemic measures, leading to less stage I cancer cases. Furthermore, excess mortality related to COVID-19 might have led to the observed slight decrease in stage IV cancer cases in 2021, as these patients were already in a weakened health condition (Federal Statistical Office 2022a).

Generally, a strong shift toward more advanced cancer cases has been reported in previous studies, such as in a study from Northern Ireland with a significant 4% points decrease in early-stage and a 2% points increase in late-stage all-cancer cases (Bennett et al. 2024) and in a study from the US with higher odds of being diagnosed with all-cancer stage IV compared to stage I to III in 2020 compared to 2019 [odds ratio = 1.074, 95% CI 1.07–1.08] (Han et al. 2023). For each of the five investigated cancer types, an upstaging during the pandemic compared to pre-pandemic has been reported in previous studies: female breast cancer (Kaltofen et al. 2022; Mentrasti et al. 2022; Heer et al. 2023; Peacock et al. 2023; Triki et al. 2024), colorectal cancer (Radulovic et al. 2021; Rottoli et al. 2022; Heer et al. 2023), prostate cancer (Heer et al. 2023), lung cancer (Melocchi et al. 2023), and skin melanoma (Davis et al. 2022; Kostner et al. 2022; Heer et al. 2023). The comparisons of cancer stage distribution between countries indicates that the Swiss healthcare system may have been less severely affected by the COVID-19 pandemic.

For all-cancer and the five most common cancer types, patients diagnosed during the pandemic received less often surgeries than those diagnosed in 2018/19. The latter trend was also observed for all-cancer patients in Brazil (-15.7% points) (Ribeiro et al. 2022) and specifically for breast cancer patients in the US (-29.6% points) (Hawrot et al. 2021) and in France (-3.5% points) (Feron Agbo et al. 2023) and lung cancer patients in northern Italy (-7.2% points) (Mangone et al. 2023). Meanwhile, in our study, patients diagnosed during the pandemic, especially all-cancer and lung cancer patients, were more often treated with immunotherapies and other treatments, including e.g., ‘active surveillance’, than those diagnosed pre-pandemic. A reason for the observed shift in treatment type might have been the increase of hospitalized patients related to COVID-19, leading to a more limited capacity of the Swiss healthcare system and therefore to less surgeries and more other treatments as initial cancer therapy (Thiabaud et al. 2021; Federal Statistical Office 2022b). Additionally, immunotherapies might have been conducted more frequently because the therapy is less affected by a limited capacity of healthcare institutions compared to surgeries and it does not supress the patient’s immune system and thereby increasing their risk for infections as a chemotherapy would do. Aligning with our reasoning, a study from northern Italy explained their observed decrease in conducted surgeries among lung cancer patients and the increase in chemotherapies with the reorganization and more limited capacity of the healthcare system related to COVID-19 (Mangone et al. 2023).

In our study, median TTI varied by incidence period, cancer type, and conducted treatment. Overall, median TTI decreased over the years and surgery revealed the lowest TTI. Especially for lung cancer and skin melanoma patients, the median TTI for surgery was often zero days. For skin melanoma, it is common that on the day of diagnosis the tumour is instantly removed by a surgery. For lung cancer patients, the diagnosis was in some cases made due to the conducted surgery and not known in advance. For instances, a lung surgery was conducted due to a different hypothesized lung disease, but the surgery then revealed the true diagnosis being lung cancer or a lung cancer diagnosis was made by detecting lung cancer metastases through a surgery. In the latter two instances, the date of diagnosis and the date of treatment start were identical and TTI was zero days.

In the Swiss cantons of ZH and ZG, generally no differences or shorter TTI were observed during the first year of the pandemic, indicating that the Swiss healthcare system managed to provide timely cancer treatments. Meanwhile, during the second pandemic year in comparison to pre-COVID-19, TTI was either similar or longer, indicating to some extent a delay in cancer treatment. In a previous study of our research group (Suter et al. 2024), an increase in cancer diagnoses in 2021 compared to 2018/19 was observed, leading to a higher demand for cancer therapies, which might explain the observed longer TTI for cancer patients diagnosed in 2021.

A study in the US on metastatic solid cancers observed no difference in median TTI and treatment selection during the pandemic (2020) compared to pre-pandemic (2019) (Parikh et al. 2022). In contrast, a study in Canada using data from 2016 to 2020 observed an overall decrease of 12.4% points in the mean TTI for all-cancer patients treated within 6-month postdiagnosis during the pandemic (35.9 days) compared to pre-pandemic (41.0 days) (Fu et al. 2023). The described trend in the latter study has been observed for patients receiving surgery, chemotherapy, and radiotherapy, indicating a general and not a therapy-specific trend (Fu et al. 2023).

For colorectal cancer, skin melanoma, and female breast cancer our study generally found no evidence for a difference in TTI among the incidence years. In line with our results, no difference in TTI was seen in the US (colorectal cancer: 2017–2019: 43 days; 2020: 42 days (Elamin et al. 2023); female breast cancer: 2018: 41 days; 2020: 36 days (Hawrot et al. 2021)), in the Netherlands (skin melanoma: March until May 2018/19: 14 days; March until May 2020 (first COVID-19 wave): 14.5 days (van Not et al. 2022)), and in France (female breast cancer: 2019: 63 days; 2020: 57 days (Feron Agbo et al. 2023)). In contrast, a study from Brazil reported shorter median TTI during COVID-19 compared to pre-COVID-19 for colorectal cancer (2018: 48 days; 2020: 35 days) and female breast cancer (2018: 48 days; 2020: 55.5 days) (Mendonça e Silva et al. 2023). Meanwhile, an increase in TTI for female breast cancer during the pandemic compared to pre-COVID-19 was observed in Tunisia (January 2018 to February 2020: 76 days; March 2020 to December 2021: 118 days) (Triki et al. 2024).

In our study, lung cancer patients receiving surgery and diagnosed in 2020 compared to 2018/19 had significantly longer and those receiving radiotherapy, chemotherapy, or other treatments shorter TTI, meanwhile those diagnosed in 2021 had shorter TTI for other treatments. The latter increase in TTI for patients diagnosed in 2020 and receiving surgery might have been caused by a more limited healthcare system capacity due to the increase of patients with a respiratory disease related to COVID-19 (Thiabaud et al. 2021; Federal Statistical Office 2022b). In a study from the Netherlands, the median TTI for lung cancer patients was even shorter during the pandemic (first wave: 48 days; between waves: 46 days; second wave: 42 days) than before the pandemic (50 days) (van Vuren et al. 2024).

In our study, prostate cancer patients generally had longer TTI in 2020 and 2021 compared to pre-COVID-19 patients, especially for surgery and other treatments. The opposite was observed in Germany (Filipas et al. 2024) and in Brazil (Mendonça e Silva et al. 2023), where studies reported a reduced median TTI during the pandemic compared to pre-pandemic. The longer TTI in Switzerland could have been due to the slight increase of prostate cancer diagnoses in 2021 compared to 2018/19 and a nearly 10% points increase from 2018/19 to 2021 of prostate cancer patients receiving other treatments.

Overall, changes in TTI varied among cancer types, countries, and incidence years.

The current study had some limitations. Our data was based on only two Swiss cantons. Therefore, the results of our study might not be generalizable to the whole country. Moreover, we investigated only the first primary treatment. Treatment combinations, neoadjuvant, adjuvant or post-operative therapies were not reflected in our study. Additionally, the data partly lacked a large enough sample size in order to conduct some therapy- and cancer type-specific regression analyses. However, the overall sample size was large and, hence, small differences in cancer outcomes can become statistically significant, but they might not be clinically significant. In 2020, i.e., in the year of the COVID-19 pandemic, a new national law on cancer registration was implemented. The influences of the new law and of the COVID-19 pandemic cannot be clearly disentangled. Lastly, the date of diagnosis represents the general detection of a tumour, but for the majority of cancer cases further medical tests (for example immunohistochemistry or Next Generation Sequencing) are needed afterwards to determine the most suitable therapy. Therefore, the calculated TTI in our study does not always represent the actual waiting time until treatment start and might be biased towards longer times. Nevertheless, the current study also had several strengths. Not only did we investigate different cancer types but also therapy specific effects which has hardly been analysed in previous studies. Furthermore, our study included two during COVID-19 years, revealing short- but also long-term effects of the pandemic on cancer outcomes. The data were population-based and provided by a cantonal cancer registry. Even though the data quality was high already before the new cancer registration law was implemented, the law ensured a complete and high-quality documentation of all cancer cases from 2020 onward. Lastly, the data of both investigated Swiss cantons was not influenced by potentially interrupted cancer screening programs and therefore, reflected a hardly investigated framework.

To conclude, less cancer diagnoses of unknown stage and slightly more diagnoses of stage II and III were observed during the pandemic compared to the pre-pandemic period. It remains debatable to what extent the pandemic and to what extent the new Swiss cancer registration law were responsible for the observed cancer stage shift. A decrease in conducted surgeries and an increase in other treatments was observed during COVID-19 compared to pre-COVID-19 years. Generally, TTI was similar or even shorter for patients diagnosed in 2020 and 2018/19, whereas those diagnosed in 2021 had partly longer TTI, representing a possible long-term effect of the COVID-19 pandemic on the Swiss public healthcare system. Our findings provide an insight into the effects of the pandemic on cancer outcomes in a hardly investigated context and may help to shape decisions of the public healthcare system during a future pandemic.

## Electronic supplementary material

Below is the link to the electronic supplementary material.

Supplementary Material 1

## Data Availability

No datasets were generated or analysed during the current study.

## Abbreviations

SARS-CoV-2: Severe Acute Respiratory Syndrome Coronavirus 2
COVID-19: Coronavirus Disease 2019
WHO: World Health Organization
ZH: Zurich
ZG: Zug
SH: Schaffhausen
SZ: Schwyz
CI: Confidence Intervals
RECORD: REporting of studies Conducted using Observational Routinely-collected Data
CRZZSS: Cancer Registry of the Cantons Zurich, Zug, Schaffhausen, and Schwyz
ICD-10: International Classification of Diseases (10th revision)
χ^2^: Chi-squared
Df: Degrees of freedom
RR: Rate Ratio
TTI: Time to Treatment Initiation
CHOP: Swiss Classification of Surgical Procedures
IQR: Interquartile range
