# Supplementary material for: Effect of the COVID-19 pandemic and lockdown on cancer stage distribution and time to treatment initiation using cancer registry data of the Swiss cantons of Zurich and Zug from 2018 to 2021
Source: J Cancer Res Clin Oncol. 2025 Feb 21;151(2):88. doi: 10.1007/s00432-025-06140-x (PMC11842471; doi:10.1007/s00432-025-06140-x)
Supplement: Supplementary file 1 — Supplementary Material 1 [file 432_2025_6140_MOESM1_ESM.docx]

**Supplementary Material**

**Effect of the COVID-19 pandemic and lockdown on cancer stage distribution and time to treatment initiation using cancer registry data of the Swiss cantons of Zurich and Zug from 2018 to 2021**

**Authors**

Flurina Suter^1,2^, Miriam Wanner^2^, Andreas Wicki^3^, Dimitri Korol^2^, Sabine Rohrmann^1,2,*^

**Affiliation**

^1^ Division of Chronic Disease Epidemiology, Epidemiology, Biostatistics and Prevention Institute (EBPI), University of Zurich, Zurich, Switzerland

^2^ Cancer Registry Zurich, Zug, Schaffhausen and Schwyz, Institute of Pathology and Molecular Pathology, University Hospital Zurich, Zurich, Switzerland

^3^ Department of Medical Oncology and Hematology, Faculty of Medicine, University and University Hospital Zurich, Zurich, Switzerland.

*Correspondence: sabine.rohrmann@uzh.ch; Tel.: +41-446-345-256

**ORCID**

Flurina Suter: 0000-0002-5302-2268

Miriam Wanner: 0000-0003-0888-0690

Andreas Wicki: 0000-0002-2924-8080

Dimitri Korol: 0000-0002-9217-8038

Sabine Rohrmann: 0000-0002-2215-1200

Table of Contents

[Fig. S1: Flow diagram of the investigated study population from 2018/19-2021 of the Swiss cantons of Zurich and Zug^1^. 4](#_Toc189659657)

[Fig. S2: Cancer stage distribution of colorectal cancer cases in the Swiss cantons of Zurich and Zug from 2018 to 2021 (n = 3291). 6](#_Toc189659658)

[Fig. S3: Cancer stage distribution of lung cancer cases in the Swiss cantons of Zurich and Zug from 2018 to 2021 (n = 3356). 7](#_Toc189659659)

[Fig. S4: Cancer stage distribution of skin melanoma cases in the Swiss cantons of Zurich and Zug from 2018 to 2021 (n = 2984). 8](#_Toc189659660)

[Fig. S5: Cancer stage distribution of female breast cancer cases in the Swiss cantons of Zurich and Zug from 2018 to 2021 (n = 4879). 9](#_Toc189659661)

[Fig. S6: Cancer stage distribution of prostate cancer cases in the Swiss cantons of Zurich and Zug from 2018 to 2021 (n = 5473). 10](#_Toc189659662)

[Fig. S7: Distribution of the time to treatment initiation stratified by incidence year and therapy for colorectal cancer cases from 2018/19 to 2021 in the cantons of Zurich and Zug (n = 2930). 11](#_Toc189659663)

[Fig. S8: Distribution of the time to treatment initiation stratified by incidence year and therapy for lung cancer cases from 2018/19 to 2021 in the cantons of Zurich and Zug (n = 2383). 12](#_Toc189659664)

[Fig. S9: Distribution of the time to treatment initiation stratified by incidence year and therapy for skin melanoma cases from 2018/19 to 2021 in the cantons of Zurich and Zug (n = 2907). 13](#_Toc189659665)

[Fig. S10: Distribution of the time to treatment initiation stratified by incidence year and therapy for female breast cancer cases from 2018/19 to 2021 in the cantons of Zurich and Zug (n = 4658). 14](#_Toc189659666)

[Fig. S11: Distribution of the time to treatment initiation stratified by incidence year and therapy for prostate cancer cases from 2018/19 to 2021 in the cantons of Zurich and Zug (n = 4354). 15](#_Toc189659667)

[Fig. S12: Quasipoisson regression models estimates and 95% confidence intervals of the time to treatment initiation stratified by incidence year and treatment group for colorectal cancer cases from 2018/19 to 2021 in the cantons of Zurich and Zug combined (n = 2884). 16](#_Toc189659668)

[Fig. S13: Quasipoisson regression models estimates and 95% confidence intervals of the time to treatment initiation stratified by incidence year and treatment group for lung cancer cases from 2018/19 to 2021 in the cantons of Zurich and Zug combined (n = 2383). 17](#_Toc189659669)

[Fig. S14: Quasipoisson regression models estimates and 95% confidence intervals of the time to treatment initiation stratified by incidence year and treatment group for skin melanoma cases from 2018/19 to 2021 in the cantons of Zurich and Zug combined (n = 2894). 18](#_Toc189659670)

[Fig. S15: Quasipoisson regression models estimates and 95% confidence intervals of the time to treatment initiation stratified by incidence year and treatment group for female breast cancer cases from 2018/19 to 2021 in the cantons of Zurich and Zug combined (n = 4600). 19](#_Toc189659671)

[Fig. S16: Quasipoisson regression models estimates and 95% confidence intervals of the time to treatment initiation stratified by incidence year and treatment group for prostate cancer cases from 2018/19 to 2021 in the cantons of Zurich and Zug combined (n = 4347). 20](#_Toc189659672)

[Fig. S17: Quasipoisson regression models estimates and 95% confidence intervals of the time to treatment initiation stratified by incidence year and treatment group for all-cancer cases from 2018/19 to 2021 in the cantons of Zurich and Zug combined with complete dates information (n = 26,993). 21](#_Toc189659673)

[Fig. S18: Quasipoisson regression models estimates and 95% confidence intervals of the time to treatment initiation stratified by incidence year and treatment group for colorectal cancer cases from 2018/19 to 2021 in the cantons of Zurich and Zug combined with complete dates information (n = 2821). 22](#_Toc189659674)

[Fig. S19: Quasipoisson regression models estimates and 95% confidence intervals of the time to treatment initiation stratified by incidence year and treatment group for lung cancer cases from 2018/19 to 2021 in the cantons of Zurich and Zug combined with complete dates information (n = 2242). 23](#_Toc189659675)

[Fig. S20: Quasipoisson regression models estimates and 95% confidence intervals of the time to treatment initiation stratified by incidence year and treatment group for skin melanoma cases from 2018/19 to 2021 in the cantons of Zurich and Zug combined with complete dates information (n = 2882). 24](#_Toc189659676)

[Fig. S21: Quasipoisson regression models estimates and 95% confidence intervals of the time to treatment initiation stratified by incidence year and treatment group for female breast cancer cases from 2018/19 to 2021 in the cantons of Zurich and Zug combined with complete dates information (n = 4459). 25](#_Toc189659677)

[Fig. S22: Quasipoisson regression models estimates and 95% confidence intervals of the time to treatment initiation stratified by incidence year and treatment group for prostate cancer cases from 2018/19 to 2021 in the cantons of Zurich and Zug combined with complete dates information (n = 4089). 26](#_Toc189659678)

[Table S1: Baseline characteristics of the study population on time to treatment initiation for the all-cancer and the five most common cancer types using data of the Swiss cantons of Zurich and Zug between 2018/19 and 2021. 27](#_Toc189659679)

[Table S2: Multivariable multinomial regression model estimates and 95% confidence interval on the analysis on stage distribution among incidence years for all-cancer and the five most common cancer types in Switzerland 28](#_Toc189659680)

[OR = Odds Ratio; CI = Confidence Interval 29](#_Toc189659681)

[Table S3: Distribution of colorectal cancer cases of the cantons of Zurich and Zug with a diagnosis from 2018/19 to 2021 stratified by incidence year and type of treatment. 29](#_Toc189659682)

[Table S4: Distribution of lung cancer cases of the cantons of Zurich and Zug with a diagnosis from 2018/19 to 2021 stratified by incidence year and type of treatment. 30](#_Toc189659683)

[Table S5: Distribution of skin melanoma cases of the cantons of Zurich and Zug with a diagnosis from 2018/19 to 2021 stratified by incidence year and type of treatment. 30](#_Toc189659684)

[Table S6: Distribution of female breast cancer cases of the cantons of Zurich and Zug with a diagnosis from 2018/19 to 2021 stratified by incidence year and type of treatment. 31](#_Toc189659685)

[Table S7: Distribution of prostate cancer cases of the cantons of Zurich and Zug with a diagnosis from 2018/19 to 2021 stratified by incidence year and type of treatment. 31](#_Toc189659686)

[References: 32](#_Toc189659687)

# **Fig. S1**: Flow diagram of the investigated study population from 2018/19-2021 of the Swiss cantons of Zurich and Zug^1^.

**Original sample size of malignant tumours (except C44) and benign brain tumours (D32-33, D43)**

**(n = 34,606)**

**Cancer Stage Analysis:**

Exclude

- Not solid cancer case^2^

(n = 4633)

Total cases^8^

(n = 29,973)

*Colorectal Cancer (n = 3291)*

*Lung Cancer (n = 3356)*

*Skin Melanoma (n = 2984)*

*Female Breast Cancer (n = 4879)*

*Prostate Cancer (n = 5473)*

**TTI Analysis:**

Exclude

- No CHOP code information on treatment (n = 4350)^3^
- Treatment documented as ‘none’ or ‘unknown’ (n = 727)^4^
- No treatment initiation date (n = 399)^5^
- TTI ≥ 300 days (n = 20)^6^
- Not first primary cancer diagnosis within 2018-2021 (n = 1027)^7^

Total cases^9^

(n = 28,083)

*Colorectal Cancer (n = 2930)*

*Lung Cancer (n = 2383)*

*Skin Melanoma (n = 2907)*

*Female Breast Cancer (n = 4658)*

*Prostate Cancer (n = 4354)*

CHOP = Swiss Classification of Surgical Procedures code; TTI = Time to Treatment Initiation;

^1^ Cancer cases were classified using the 10^th^ revision of the international classification of diseases (ICD-10) code: Colorectal cancer: ICD-10 C18-C20; Lung cancer: ICD-10 C34; Skin melanoma: ICD-10 C43; Female breast cancer: ICD-10 C50; Prostate cancer: ICD-10 C61 (World Health Organization 2019).

^2^ Not solid cancer cases were excluded because for these cancer types no staging information was assessed or another staging coding system than the one for solid cancer cases was applied. These not solid cancer types included cancer diagnosis with the following ICD-10 codes (n = 4568): D32-D33 (n = 763), D43 (n = 94), C49 (n = 204), C70-C72 (n = 511), C74-C75 (n = 51), C81-C86 (n = 1383), C88 (n = 198), C90-C96 (n = 1364). Additionally, we excluded patients with stage information coded as ‘not applicable’ (n = 65): ICD-10 C14 (n = 1), C22 (n = 1), C25 (n = 1), C30 (n = 1), C32 (n = 2), C33 (n = 1), C34 (n = 4), C38 (n = 3), C41 (n = 5), C45 (n = 5), C46 (n = 17), C47 (n = 2), C48 (n = 1), C50 (n = 8), C56 (n = 1), C62 (n = 1), C63 (n = 1), C64 (n = 2), C69 (n = 7), C73 (n = 1).

^3^ If no CHOP code on treatment information was available, the cancer case was excluded, because the type of treatment could not be determined and categorized (n = 4350). These included 3913 cases diagnosed in 2018/2019, 272 cases diagnosed in 2020, and 165 cases diagnosed in 2021.

^4^ If the treatment was categorized as ‘none’ or ‘unknown’ the cancer case was excluded because the case could not be categorized to a pre-specified treatment group (n = 727). These included 74 cases diagnosed in 2018/2019, 234 cases diagnosed in 2020, and 419 cases diagnosed in 2021.

^5^ If the date of treatment initiation was missing, the TTI could not be determined. Therefore, these cases were excluded for the TTI analysis (n = 399). These included 244 cases diagnosed in 2018/2019, 106 cases diagnosed in 2020, and 49 cases diagnosed in 2021.

^6^ Patients with longer TTI than 300 days were excluded, because the long TTI has been most probably due to a personal reason, e.g., no treatment wanted, or due to a medical reason, e.g., treating of a following primary cancer diagnoses had priority, rather than due to the COVID-19 pandemic. Including these patients would lead to biased results and therefore, they were excluded (n = 20). Of these cases 6 were diagnosed in 2018/2019, 7 in 2020, and 7 in 2021.

^7^ If the registered cancer diagnosis was not the first primary cancer diagnosis within the investigated period (2018-2021), the cancer case was excluded (n = 1027), because TTI is most probably dependent on the treatment status of the other primary cancer diagnosis. Therefore, including these cancer cases would lead to biased TTI. Of these cases 298 were diagnosed in 2018/2019, 338 in 2020, and 391 in 2021.

^8^ After applying the exclusion criteria, the final study population of the cancer stage analysis consisted of cancer cases of the following ICD-10 codes: C00-C26, C30-C34, C37-C41, C43, C45-C48, C50-C58, C60-C69, C73, C76, C80.

^9^ After applying the exclusion criteria, the final study population of the TTI analysis consisted of cancer cases of the following ICD-10 codes: C00-C26, C30-C34, C37-C41, C43, C45-C58, C60-C76, C80-C86, C88, C90-C96, D32-D33, D43.

# **Fig. S2**: Cancer stage distribution of colorectal cancer cases in the Swiss cantons of Zurich and Zug from 2018 to 2021 (n = 3291).

# **Fig. S3**: Cancer stage distribution of lung cancer cases in the Swiss cantons of Zurich and Zug from 2018 to 2021 (n = 3356).

# **Fig. S4**: Cancer stage distribution of skin melanoma cases in the Swiss cantons of Zurich and Zug from 2018 to 2021 (n = 2984).

# **Fig. S5**: Cancer stage distribution of female breast cancer cases in the Swiss cantons of Zurich and Zug from 2018 to 2021 (n = 4879).

# **Fig. S6**: Cancer stage distribution of prostate cancer cases in the Swiss cantons of Zurich and Zug from 2018 to 2021 (n = 5473).

# **Fig. S7**: Distribution of the time to treatment initiation stratified by incidence year and therapy for colorectal cancer cases from 2018/19 to 2021 in the cantons of Zurich and Zug (n = 2930).

# **Fig. S8**: Distribution of the time to treatment initiation stratified by incidence year and therapy for lung cancer cases from 2018/19 to 2021 in the cantons of Zurich and Zug (n = 2383).

# **Fig. S9**: Distribution of the time to treatment initiation stratified by incidence year and therapy for skin melanoma cases from 2018/19 to 2021 in the cantons of Zurich and Zug (n = 2907).

# **Fig. S10**: Distribution of the time to treatment initiation stratified by incidence year and therapy for female breast cancer cases from 2018/19 to 2021 in the cantons of Zurich and Zug (n = 4658).

# **Fig. S11**: Distribution of the time to treatment initiation stratified by incidence year and therapy for prostate cancer cases from 2018/19 to 2021 in the cantons of Zurich and Zug (n = 4354).

# **Fig. S12**: Quasipoisson regression models estimates and 95% confidence intervals of the time to treatment initiation stratified by incidence year and treatment group for colorectal cancer cases from 2018/19 to 2021 in the cantons of Zurich and Zug combined (n = 2884).

# **Fig. S13:** Quasipoisson regression models estimates and 95% confidence intervals of the time to treatment initiation stratified by incidence year and treatment group for lung cancer cases from 2018/19 to 2021 in the cantons of Zurich and Zug combined (n = 2383).

# **Fig. S14**: Quasipoisson regression models estimates and 95% confidence intervals of the time to treatment initiation stratified by incidence year and treatment group for skin melanoma cases from 2018/19 to 2021 in the cantons of Zurich and Zug combined (n = 2894).

**Fig. S15**: Quasipoisson regression models estimates and 95% confidence intervals of the time to treatment initiation stratified by incidence year and treatment group for female breast cancer cases from 2018/19 to 2021 in the cantons of Zurich and Zug combined (n = 4600).

# **Fig. S16**: Quasipoisson regression models estimates and 95% confidence intervals of the time to treatment initiation stratified by incidence year and treatment group for prostate cancer cases from 2018/19 to 2021 in the cantons of Zurich and Zug combined (n = 4347).

# **Fig. S17**: Quasipoisson regression models estimates and 95% confidence intervals of the time to treatment initiation stratified by incidence year and treatment group for all-cancer cases from 2018/19 to 2021 in the cantons of Zurich and Zug combined with complete dates information (n = 26,993).

# **Fig. S18**: Quasipoisson regression models estimates and 95% confidence intervals of the time to treatment initiation stratified by incidence year and treatment group for colorectal cancer cases from 2018/19 to 2021 in the cantons of Zurich and Zug combined with complete dates information (n = 2821).

# **Fig. S19**: Quasipoisson regression models estimates and 95% confidence intervals of the time to treatment initiation stratified by incidence year and treatment group for lung cancer cases from 2018/19 to 2021 in the cantons of Zurich and Zug combined with complete dates information (n = 2242).

# **Fig. S20**: Quasipoisson regression models estimates and 95% confidence intervals of the time to treatment initiation stratified by incidence year and treatment group for skin melanoma cases from 2018/19 to 2021 in the cantons of Zurich and Zug combined with complete dates information (n = 2882).

# **Fig. S21**: Quasipoisson regression models estimates and 95% confidence intervals of the time to treatment initiation stratified by incidence year and treatment group for female breast cancer cases from 2018/19 to 2021 in the cantons of Zurich and Zug combined with complete dates information (n = 4459).

# **Fig. S22**: Quasipoisson regression models estimates and 95% confidence intervals of the time to treatment initiation stratified by incidence year and treatment group for prostate cancer cases from 2018/19 to 2021 in the cantons of Zurich and Zug combined with complete dates information (n = 4089).

# **Table S1**: Baseline characteristics of the study population on time to treatment initiation for the all-cancer and the five most common cancer types using data of the Swiss cantons of Zurich and Zug between 2018/19 and 2021.

| **Variables** | **All-cancer^a^** | **Colorectal Cancer^a^** | **Lung Cancer^a^** | **Skin Melanoma^a^** | **Female Breast Cancer^a^** | **Prostate Cancer^a^** |
| --- | --- | --- | --- | --- | --- | --- |
| **n**  **Sex**, n (%)  Males  Females | 28,083  14,450 (51.5%)  13,633 (48.5%) | 2930  1516 (51.7%)  1414 (48.3%) | 2383  1314 (55.1%)  1069 (44.9%) | 2907  1566 (53.9%)  1341 (46.1%) | 4658  0 (0.0%)  4658 (100.0%) | 4354  4354 (100.0%)  0 (0.0%) |
| **Age at Incidence** (median [IQR])  **Canton**, n (%)  Zurich (ZH)  Zug (ZG) | 67.0 [56.0, 76.0]  25,973 (92.5%)  2110 (7.5%) | 71.0 [59.0, 79.0]  2689 (91.8%)  241 (8.2%) | 69.0 [62.0, 76.0]  2213 (92.9%)  170 (7.1%) | 66.0 [52.0, 77.0]  2630 (90.5%)  277 (9.5%) | 62.0 [51.0, 74.0]  4279 (91.9%)  379 (8.1%) | 70.0 [63.0, 75.0]  4063 (93.3%)  291 (6.7%) |
| **Incidence Year**, n (%)  2018-2019  2020  2021 | 12,635 (45.0%)  7469 (26.6%)  7979 (28.4%) | 1456 (49.7%)  717 (24.5%)  757 (25.8%) | 1005 (42.2%)  707 (29.7%)  671 (28.2%) | 1448 (49.8%)  692 (23.8%)  767 (26.4%) | 2339 (50.2%)  1111 (23.9%)  1208 (25.9%) | 1716 (39.4%)  1222 (28.1%)  1416 (32.5%) |

^a^ Cancer cases were defined using the 10^th^ revision of the international classification of diseases (ICD-10): All-cancer: all malignant cancers (except C44) and benign brain cancer (ICD-10: D32-33, D43); Colorectal cancer: ICD-10 C18-C20; Lung cancer: ICD-10 C34; Skin melanoma: ICD-10 C43; Female breast cancer: ICD-10 C50; Prostate cancer: ICD-10 C61 (World Health Organization 2019).

**Table S2**: Multivariable multinomial regression model estimates and 95% confidence interval on the analysis on stage distribution among incidence years for all-cancer and the five most common cancer types in Switzerland^a^

| **Cancer Group^b^** | | **Cancer Stage** | | | |
| --- | --- | --- | --- | --- | --- |
|  |  | **Stage II**  OR [95% CI] | **Stage III**  OR [95% CI] | **Stage IV**  OR [95% CI] | **Stage unknown**  OR [95% CI] |
| **All-Cancer** | **Incidence Year**  2018/2019 (ref.)  2020  2021 | 1.00 [-]  1.15 [1.07; 1.25]  1.14 [1.05; 1.23] | 1.00 [-]  1.25 [1.15; 1.37]  1.13 [1.03; 1.22] | 1.00 [-]  1.08 [1.00; 1.17]  0.97 [0.90; 1.05] | 1.00 [-]  0.31 [0.27; 0.35]  0.25 [0.22; 0.29] |
| **Colorectal Cancer** | **Incidence Year**  2018/2019 (ref.)  2020  2021 | 1.00 [-]  0.89 [0.69; 1.14]  0.98 [0.77; 1.25] | 1.00 [-]  1.26 [0.99; 1.60]  1.14 [0.89; 1.45] | 1.00 [-]  0.84 [0.64; 1.10]  0.94 [0.72; 1.21] | 1.00 [-]  0.25 [0.17; 0.38]  0.30 [0.20; 0.44] |
| **Lung Cancer** | **Incidence Year**  2018/2019 (ref.)  2020  2021 | 1.00 [-]  0.80 [0.56; 1.14]  0.69 [0.48; 1.00] | 1.00 [-]  0.87 [0.66; 1.15]  0.81 [0.62; 1.06] | 1.00 [-]  0.92 [0.73; 1.15]  0.76 [0.61; 0.95] | 1.00 [-]  0.11 [0.06; 0.19]  0.23 [0.15; 0.35] |
| **Skin Melanoma** | **Incidence Year**  2018/2019 (ref.)  2020  2021 | 1.00 [-]  0.81 [0.55; 1.19]  0.96 [0.68; 1.35] | 1.00 [-]  1.08 [0.73; 1.59]  0.95 [0.64; 1.40] | 1.00 [-]  1.04 [0.58; 1.85]  0.94 [0.54; 1.64] | 1.00 [-]  0.93 [0.65; 1.33]  0.49 [0.33; 0.74] |
| **Female Breast Cancer** | **Incidence Year**  2018/2019 (ref.)  2020  2021 | 1.00 [-]  1.19 [1.02; 1.40]  1.16 [1.00; 1.36] | 1.00 [-]  1.13 [0.89; 1.43]  0.99 [0.78; 1.25] | 1.00 [-]  1.29 [0.98; 1.68]  1.04 [0.79; 1.36] | 1.00 [-]  0.62 [0.33; 1.16]  0.64 [0.36; 1.14] |
| **Prostate Cancer** | **Incidence Year**  2018/2019 (ref.)  2020  2021 | 1.00 [-]  1.89 [1.59; 2.25]  1.65 [1.41; 1.94] | 1.00 [-]  1.54 [1.26; 1.90]  1.34 [1.10; 1.62] | 1.00 [-]  1.74 [1.42; 2.12]  1.53 [1.27; 1.85] | 1.00 [-]  0.52 [0.35; 0.78]  0.37 [0.24; 0.56] |

# OR = Odds Ratio; CI = Confidence Interval

^a^ The model was adjusted for incidence year (2018/19, 2020, 2021), incidence month (January to December), age group (<60, 60-69, 70-79, >=80), sex (males, females; only for all-cancer, colorectal cancer, lung cancer, and skin melanoma analysis), and canton of resident (Zurich, Zug). The reference outcome category were the stage I cancer cases.

^b^ Cancer cases were defined using the 10th revision of the international classification of diseases (ICD-10): All-cancer: all malignant cancers (except C44) and benign brain cancer (ICD-10: D32-33, D43); Colorectal cancer: ICD-10 C18-C20; Lung cancer: ICD-10 C34; Skin melanoma: ICD-10 C43; Female breast cancer: ICD-10 C50; Prostate cancer: ICD-10 C61 (World Health Organization 2019).

# **Table S3**: Distribution of colorectal cancer cases of the cantons of Zurich and Zug with a diagnosis from 2018/19 to 2021 stratified by incidence year and type of treatment.

|  | **Year of cancer diagnosis** | | |
| --- | --- | --- | --- |
|  | **2018/19^a^**  **(n = 728)** | **2020**  **(n = 717)** | **2021**  **(n = 757)** |
| **Type of treatment,** n (%)  Surgery  Radiotherapy  Chemotherapy  Immunotherapy  Hormone therapy  Stem cell transplant  Other treatment | 573 (78.6%)  104 (14.2%)  52 (7.1%)  0 (0.0%)  1 (0.1%)  0 (0.0%)  0 (0.0%) | 526 (73.4%)  120 (16.7%)  47 (6.6%)  0 (0.0%)  0 (0.0%)  0 (0.0%)  24 (3.3%) | 546 (72.1%)  131 (17.3%)  59 (7.8%)  2 (0.3%)  0 (0.0%)  0 (0.0%)  19 (2.5%) |

^a^ Average absolute number and percentage of diagnosis from 2018 to 2019.

# **Table S4**: Distribution of lung cancer cases of the cantons of Zurich and Zug with a diagnosis from 2018/19 to 2021 stratified by incidence year and type of treatment.

|  | **Year of cancer diagnosis** | | |
| --- | --- | --- | --- |
|  | **2018/19^a^**  **(n = 503)** | **2020**  **(n = 707)** | **2021**  **(n = 671)** |
| **Type of treatment,** n (%)  Surgery  Radiotherapy  Chemotherapy  Immunotherapy  Hormone therapy  Stem cell transplant  Other treatment | 228 (45.3%)  99 (19.6%)  153 (30.4%)  15 (2.9%)  0 (0.0%)  0 (0.0%)  9 (1.8%) | 219 (31.0%)  131 (18.5%)  217 (30.7%)  62 (8.8%)  0 (0.0%)  0 (0.0%)  78 (11.0%) | 236 (35.2%)  106 (15.8%)  184 (27.4%)  76 (11.3%)  0 (0.0%)  0 (0.0%)  69 (10.3%) |

^a^ Average absolute number and percentage of diagnosis from 2018 to 2019.

# **Table S5**: Distribution of skin melanoma cases of the cantons of Zurich and Zug with a diagnosis from 2018/19 to 2021 stratified by incidence year and type of treatment.

|  | **Year of cancer diagnosis** | | |
| --- | --- | --- | --- |
|  | **2018/19^a^**  **(n = 724)** | **2020**  **(n = 692)** | **2021**  **(n = 767)** |
| **Type of treatment,** n (%)  Surgery  Radiotherapy  Chemotherapy  Immunotherapy  Hormone therapy  Stem cell transplant  Other treatment | 723 (99.9%)  0 (0.0%)  1 (0.1%)  1 (0.1%)  0 (0.0%)  0 (0.0%)  0 (0.0%) | 686 (99.1%)  2 (0.3%)  0 (0.0%)  2 (0.3%)  0 (0.0%)  0 (0.0%)  2 (0.3%) | 762 (99.3%)  0 (0.0%)  0 (0.0%)  3 (0.4%)  0 (0.0%)  0 (0.0%)  2 (0.3%) |

^a^ Average absolute number and percentage of diagnosis from 2018 to 2019.

# **Table S6**: Distribution of female breast cancer cases of the cantons of Zurich and Zug with a diagnosis from 2018/19 to 2021 stratified by incidence year and type of treatment.

|  | **Year of cancer diagnosis** | | |
| --- | --- | --- | --- |
|  | **2018/19^a^**  **(n = 1170)** | **2020**  **(n = 1111)** | **2021**  **(n = 1208)** |
| **Type of treatment,** n (%)  Surgery  Radiotherapy  Chemotherapy  Immunotherapy  Hormone therapy  Stem cell transplant  Other treatment | 898 (76.8%)  11 (0.9%)  178 (15.2%)  4 (0.2%)  78 (6.6%)  0 (0.0%)  4 (0.3%) | 801 (72.1%)  10 (0.9%)  197 (17.7%)  9 (0.8%)  88 (7.9%)  0 (0.0%)  6 (0.5%) | 887 (73.4%)  13 (1.1%)  187 (15.5%)  24 (2.0%)  89 (7.4%)  0 (0.0%)  8 (0.7%) |

^a^ Average absolute number and percentage of diagnosis from 2018 to 2019.

# **Table S7**: Distribution of prostate cancer cases of the cantons of Zurich and Zug with a diagnosis from 2018/19 to 2021 stratified by incidence year and type of treatment.

|  | **Year of cancer diagnosis** | | |
| --- | --- | --- | --- |
|  | **2018/19^a^**  **(n = 858)** | **2020**  **(n = 1222)** | **2021**  **(n = 1416)** |
| **Type of treatment,** n (%)  Surgery  Radiotherapy  Chemotherapy  Immunotherapy  Hormone therapy  Stem cell transplant  Other treatment | 704 (82.1%)  22 (2.6%)  3 (0.3%)  0 (0.0%)  88 (10.3%)  0 (0.0%)  42 (4.8%) | 715 (58.5%)  72 (5.9%)  1 (0.1%)  0 (0.0%)  234 (19.1%)  0 (0.0%)  200 (16.4%) | 854 (60.3%)  95 (6.7%)  1 (0.1%)  0 (0.0%)  254 (17.9%)  0 (0.0%)  212 (15.0%) |

^a^ Average absolute number and percentage of diagnosis from 2018 to 2019.

# **References:**

World Health Organization (2019) ICD-10 version: 2019 - International statistical classification of diseases and related health problems 10th revision. https://icd.who.int/browse10/2019/en. Accessed 18 Nov 2022
